# Supplementary material for: Measuring Satisfaction and Comfort with Gender Identity and Gender Expression among Transgender Women: Development and Validation of the Psychological Gender Affirmation Scale
Source: Int J Environ Res Public Health. 2021 Mar 23;18(6):3298. doi: 10.3390/ijerph18063298 (PMC8005192; doi:10.3390/ijerph18063298)
Supplement: Supplementary file 1 [file ijerph-18-03298-s001.pdf]

Supplementary Figure 1: Flowchart of participant inclusion

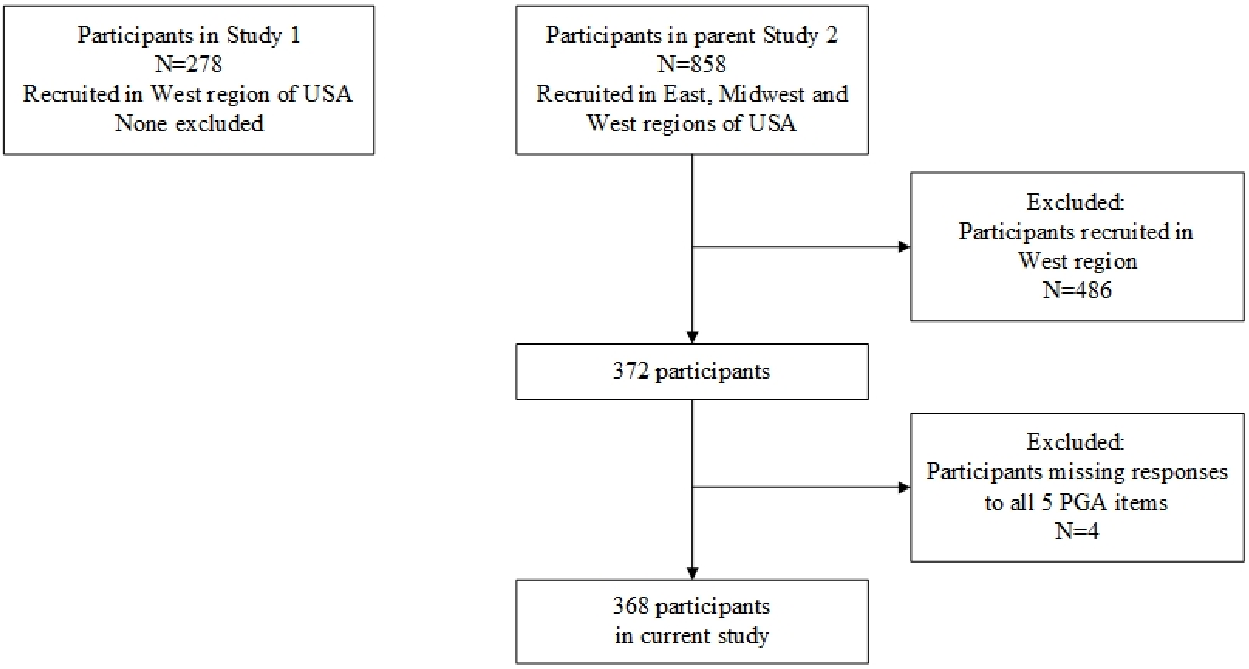

Supplementary Table 1: Descriptive characteristics of participants in Study 1 and Study 2 by geographic region of United States of America

**Characteristic**	**Study 1**		**Study 2**			
	West (N=278)		East (N=167)		Midwest (N=201)	
Age in years - mean (std. dev)	43.5	(10.7)	36.1	(10.8)	32.6	(10.6)
	n	(%)	n	(%)	n	(%)
Race-Ethnicity						
Hispanic, Latina, or of Spanish origin	91	(32.7)	141	(84.4)	23	(11.4)
Black, non-Hispanic	126	(45.3)	16	(9.6)	171	(85.1)
White, non-Hispanic	19	(6.8)	-	-	-	-
Asian or Pacific Islander, non-Hispanic	8	(2.9)	0	(0)	1	(0.5)
Additional, non-Hispanic	3	(1.1)	1	(0.6)	0	(0)
Multiracial, non-Hispanic	30	(10.8)	1	(0.6)	4	(2.0)
No response	1	(0.4)	8	(4.8)	2	(1.0)
Education						
Less than grade 12	78	(28.1)	76	(45.5)	45	(22.4)
Grade 12	109	(39.2)	44	(26.4)	99	(49.3)

Some college or higher	91	(32.8)	33	(19.8)	54	(26.9)
No response	0	(0)	14	(8.4)	3	(1.5)
Financially secure[1]	49	(17.6)	38	(22.8)	50	(24.9)
Experienced homelessness in previous 6 months	114	(41.0)	67	(40.1)	96	(47.8)
Sex work as a source of income in previous 6 months	50	(18.0)	44	(26.4)	90	(44.8)
Currently taking hormones	187	(67.3)	69	(41.3)	104	(51.7)

[1] Study 1:'Currently', Study 2: 'in the previous 6 months'
